# An unusual cause of bowel obstruction: Rhizopus Arrhizus diverticulitis

**DOI:** 10.1016/j.mmcr.2019.06.004

**Published:** 2019-06-08

**Authors:** Chungting Kou, Matthew Rendo, David Kline, Bradie Bishop, Heather C. Yun

**Affiliations:** a Department of Internal Medicine, Brooke Army Medical Center, 3551 Roger Brooke Drive, San Antonio, TX, 78234, United States; b Infectious Disease Service, Brooke Army Medical Center, 3551 Roger Brooke Drive, San Antonio, TX, 78234, United States; c Uniformed Services University of the Health Sciences, Brooke Army Medical Center, 3551 Roger Brooke Drive, San Antonio, TX, 78234, United States; d Department of Pathology, Brooke Army Medical Center, 3551 Roger Brooke Drive, San Antonio, TX, 78234, United States

**Keywords:** Mucormycosis, Rhizopus arrhizus, Fungal diverticulitis, Salvage isavuconazole

## Abstract

Mucormycosis is a fungal infection primarily afflicting immunocompromised or diabetic patients. Its presentation ranges from rhino-orbito-cerebral infections to disseminated mucormycosis with angioinvasion. We present a patient who developed a bowel obstruction one month after bone marrow transplant and was diagnosed with *Rhizopus arrhizus* diverticulitis despite antifungal therapy since transplantation. She underwent surgical removal with immediate fungal resurgence, declined further invasive intervention and was discharged on palliative isavuconazole. Seven months later she is alive with fungal containment.

## 1 Introduction

Mucormycosis is a rare but serious fungal infection caused by environmentally ubiquitous molds. While it is classically associated with infections of the head, sinuses, orbits and cerebrum, it can affect any part of the body and may be found in the wounds of trauma patients. Infection occurs primarily in immunocompromised and/or diabetic patients [1]. Mucormycosis frequently presents with nonspecific symptoms which may lead to delayed diagnosis and treatment resulting in higher mortality [2]. Endorsed clinical management guidance is limited to the 2013 European Society for Clinical Microbiology, Infectious Diseases (ESCMID) guidelines, and the cited level of evidence is frequently limited to retrospective reviews, pre-clinical animal studies and expert opinion [3,4]. We present an uncommon presentation of mucormycosis and to date its first description of gastrointestinal mucormycosis described with effective salvage isavuconazole.

## 2 Case

**Image 1:**
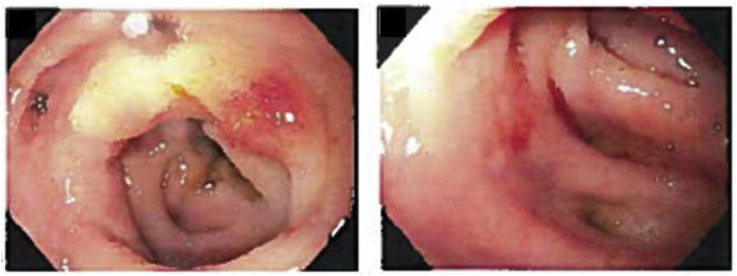
Initial sigmoidoscopy revealing diverticulosis and little sigmoid inflammation.

**Image 2:**
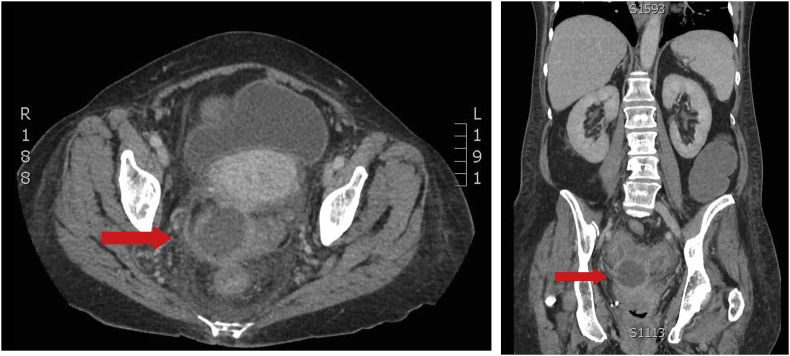
CT imaging of the abdomen revealing a 4.9cm thick-walled focal fluid collection between the posterior wall of the uterus and redundant sigmoid colon concerning for perirectal abscess.

**Image 3:**
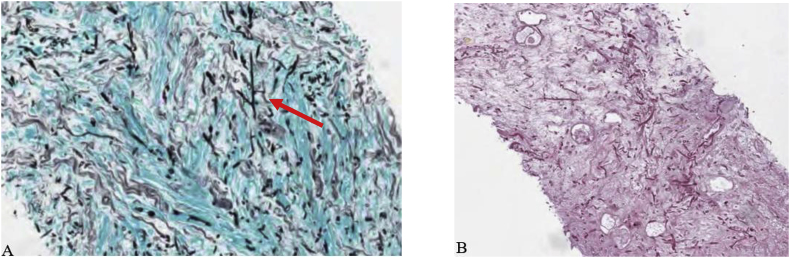
Abscess aspirate revealed large numbers of fungal elements with almost no inflammatory response. Exhibit A: GMS stain highlighting acute branching fungal elements in bowel wall. Exhibit B: core biopsy of bowel with branching fungus in a background of necrotic small bowel.

We report a sixty-two year old female with myelodysplastic syndrome that converted to acute myeloid leukemia. After initiation of induction chemotherapy with Vyxeos, she developed neutropenic fever with enteritis for which she underwent sigmoidoscopy on cycle 1 day 35 of Vyxeos which showed diverticulosis (Image 1). Biopsy revealed no fungal elements and minimal inflammation. She was readmitted approximately one month later for haploidentical stem cell transplantation (HSCT). Prophylactic posaconazole started on day +5 post HSCT. Her hospital course was prolonged due to delayed engraftment with ongoing neutropenia. Four weeks post-transplant she started experiencing abdominal pain and decreased stool output on day +26. Abdominal computed tomography (CT) demonstrated a 4.9 cm perirectal abscess on day +30 (Image 2). Cytology of the abscess aspirate revealed aseptate fungal hyphae concerning for mucormycosis (Image 3). (1,3)-β-D-glucan and galactomannan assays were negative. Her absolute neutrophil count had begun to increase up to 900 and she had been taking prophylactic posaconazole prior to HSCT. CT imaging of the sinuses, head and chest were obtained and revealed no other foci of infection. Liposomal amphotericin B at 5mg/kg/day intravenously was started on day +30 with cessation of posaconazole on day +30. Additionally, micafungin 100mg intravenously daily was initiated on day +34.

Fungal isolate from the fungal culture was sent to the University of Texas Health Science Center at San Antonio Fungus Testing Laboratory for species identification. For molecular identification, portions of the culture were suspended in Buffer G2 (Qiagen, Valencia, CA) followed by lysing using a bead beater instrument (Precellys Evolution, Bertin Instruments, Rockville, MD). Proteinase K was added, incubation occurred at 56 °C, and the DNA was extracted using an EZ1 DNA tissue kit with a BioRobot EZ1 instrument (Qiagen). The internal transcribed spacer region (ITS) and D1/D2 rRNA gene were then amplified by polymerase chase reaction using previously described primers [[5], [6], [7]]. The PCR products were sequenced, assembled, and analyzed using Sequencher software version 5.4.6 (Gene Codes, Ann Arbor, MI), and the sequences were queried in GenBank using the BLASTn algorithm at the NCBI website (www.ncbi.nlm.nih.gov). The ITS sequence demonstrated 100% identity to *Rhizopus arrhizus* (GenBank Accession No. AY213684; base pair match 570/570), and the D1/D2 sequence also showed 100% identity to *Rhizopus arrhizus* (GenBank Accession No. AY213624; base pair match 636/636). Further barcode analysis of the ITS sequence demonstrated that the isolate was *Rhizopus arrhizus* var. *arrhizus* [8].

**Image 4:**
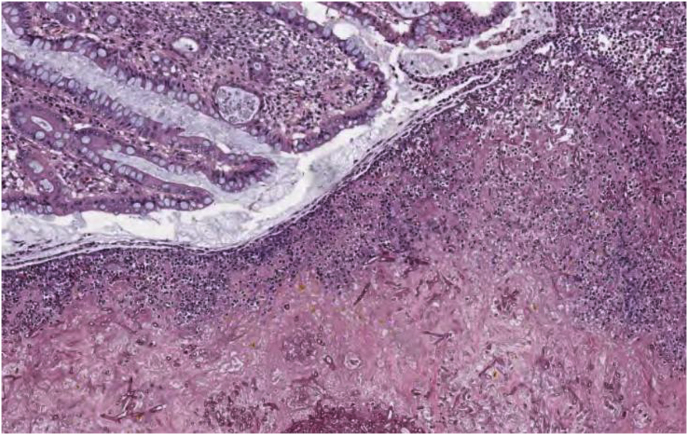
Colonic segment surgically removed revealed full thickness tissue necrosis with numerous fungal elements branching at acute angles and mixed inflammation including foreign body giant cell reaction associated with diverticula.

**Image 5:**
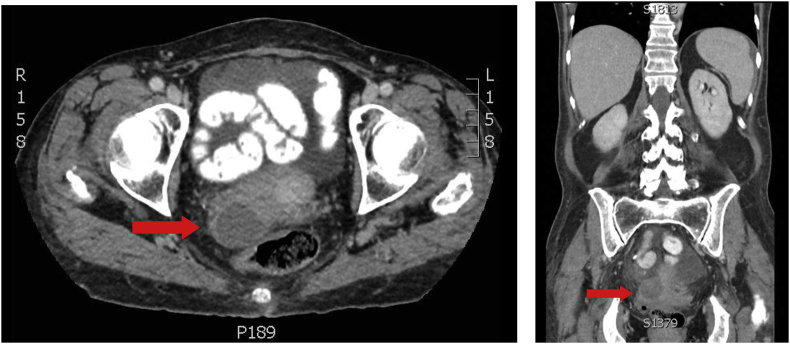
Repeat CT imaging of the abdomen and pelvis four months after surgical revision and abscess reformation shows stable complex fluid collection at 3.2 × 3.0cm from 3.0 × 2.9cm four months prior.

The patient underwent a proctosigmoidectomy with colostomy to remove the fungal abscess on day +33. The post-operative diagnosis was fungal diverticulitis (Image 4). However, six days (day+39) following surgery she had return of fevers and abdominal pain. Repeat abdominal CT showed recurrence of the fungal abscess despite surgical excision and continued antifungal therapy. Given the high likelihood of poor surgical outcome for repeat debridement and likely need for pelvic evisceration, the patient declined further intervention. She was discharged to hospice with palliative isavuconazole on day +40. She was loaded with 6 doses oral isavuconazole 375mg every 8 hours followed by daily oral isavuconazole 375mg. However, the patient remained clinically stable on isavuconazole, and repeat imaging 4 months (day + 105) following discharge demonstrated interval improvement of the abscess though without complete resolution (Image 5). The patient is currently clinically well and on hospice 7 months after her *Rhizopus Arrhizus* diverticulitis diagnosis and routinely followed in the outpatient bone marrow transplant clinic without serum isavuconazole level checks.

## 3 Discussion

Mucormycosis is a feared fungal infection often affecting immunocompromised or diabetic patients with high morbidity and mortality. While rhino-cerebro-orbital and pulmonary infections are most frequently encountered, other manifestations may present as our case demonstrates [1]. Described primarily in neonates and infants with gastric infection or necrotizing enterocolitis, gastrointestinal mucormycosis has also been described in adults [9]. It is one of the rarer manifestations, representing only 7–8% of all mucormycosis cases [1,10]. Antemortem diagnosis is made in 25% of cases, and mortality may reach as high as 85% largely due to delay in diagnosis [11].

Clinical manifestations of gastrointestinal mucormycosis are diverse, nonspecific, and range from nausea, vomiting, diarrhea and anorexia to neutropenic fever with obstruction, perforation, peritonitis, sepsis and rapid demise. Mucosal ulceration with hemorrhage is also a well-documented finding along with adjacent demarcated necrosis, edema and vascular thrombosis which is seen on endoscopy or post-operative specimens. Histopathology notes aseptate hyphae with angioinvasion, infarction and necrosis [12]. The stomach is most predominately affected followed by the colon and the ileum [13]. Dissemination to liver, pancreas and other organs is possible [14]. Colonic mucormycosis is frequently right-sided, as one case series noted in 8 of 12 colonic infections between 1985 and 2006, and can be confused clinically with typhlitis in the neutropenic patient [12]. Mucorales appendicitis has been described, though we could find only a single other case of fungal diverticulitis in the literature [15,16].

Risk factors for gastrointestinal mucormycosis include immunocompromise, malnutrition and prematurity in neonates, steroid and biologics use for immunosuppression as well as ameboid colitis, typhoid and pellagra [12,14]. Route of entry is assumed to be ingestion or exposure to contaminated material within and outside of the hospital [4,14]. With recent sigmoidoscopy and biopsy in the preceding month, peri-procedural fungal acquisition may have led to the patient's presentation.

Currently, no blood antigen tests are widely available in order to rapidly detect mucormycosis and few labs keep *Rhizopus arrhizus* primers on hand [4]. Definitive diagnosis requires recovery of fungal elements from histology or culture. Delays in antifungal therapy are associated with increased mortality [2]. Thus, early recognition of gastrointestinal mucormycosis is imperative for optimal outcomes.

High quality evidence regarding optimal antifungal therapy regimens is lacking, though the available ESCMID guidelines recommend combined surgical debridement and liposomal amphotericin B at 5 mg/kg daily [3]. A combined surgical and medical approach is recommended due to the high risk of gut perforation associated with gastrointestinal mucormycosis [4,9,17]. Salvage therapy with oral posaconazole is recommended in refractory disease or amphotericin intolerance [4]. Because our patient had breakthrough mucormycosis while on posaconazole, she was initiated on combination amphotericin B and an echinocandin. Clinical data on combination therapy using an echinocandin is limited. While small retrospective studies and pre-clinical animal data are supportive of combination therapy, no benefit has been noted in larger, more recent retrospective reviews [[18], [19], [20], [21], [22], [23]]. Despite surgery and combination antifungal therapy, our patient had refractory pelvic mucormycosis and was transitioned to salvage isavuconazole which has thus far controlled her infection.

Our case represents a rare manifestation of gastrointestinal mucormycosis: *Rhizopus arrhizus* diverticulitis resulting in bowel obstruction. Additionally, it supports the use of isavuconazole as salvage therapy for amphotericin-intolerant or refractory infection. Regardless of site of infection, further diagnostic and therapeutic innovations are needed to improve outcomes. In the meantime, improvement in mortality relies on high index of suspension and early management.

## Conflict of interest

The view(s) expressed herein are those of the author(s) and do not reflect the official policy or position of Brooke Army Medical Center, the Department of Defense, the U.S. Air Force, the U.S. Army, the U.S. Air Force Medical Department, the U.S. Army Medical Department, the United States Air Force Office of the Surgeon General, the United States Army Office of the Surgeon General or the U.S. Government.

## No conflicts of interest

There are no conflicts of interest for any authors.
